# Electrophysiological underpinnings of dysfunctional inhibitory control in adults with attention-deficit/hyperactivity disorder: evidence for reduced NoGo anteriorization

**DOI:** 10.1007/s00702-023-02639-0

**Published:** 2023-05-02

**Authors:** Szilvia Papp, László Tombor, Brigitta Kakuszi, János M. Réthelyi, István Bitter, Pál Czobor

**Affiliations:** Department of Psychiatry and Psychotherapy, Semmelweis University, Balassa Utca 6., Budapest, 1083 Hungary

**Keywords:** EEG, GFP, NoGo anteriorization, Adult ADHD, Inhibition

## Abstract

Our aim was to delineate the electrophysiological basis of dysfunctional inhibitory control of adult ADHD via investigating the anteriorization of the P3 component of the event-related brain response associated with the NoGo task condition (i.e., NoGo anteriorization, NGA). NGA is a neurophysiological measure of brain topography for cognitive response control, which indexes an overall shift of the brain’s electrical activity in anterior direction towards the prefrontal areas. While the NoGo P3 received considerable attention in the adult ADHD literature, the brain topography of this component, which reflects the inhibitory process, remains largely unaddressed. EEG recordings were obtained during a Go/NoGo task from 51 subjects (*n* = 26 adult patients with ADHD, *n* = 25 healthy controls) using a high-density, 128-channel BioSemi ActiveTwo recording system. ADHD patients had significantly lower P3 NGA response compared to controls. The decrease in NGA was related to impulsivity scores as measured by the Conners’ Adult ADHD Rating Scale: patients with higher impulsivity scores had significantly lower NGA. Treatment with stimulant medication, as compared to the lack of such treatment, was associated with a correction of the lower NGA response in ADHD patients. The current study revealed a lower NGA in adult ADHD, a finding which is consistent with the inhibitory control and frontal lobe dysfunctions described in the disorder. Our finding of the inverse relationship between NGA and impulsivity suggests that clinically more severe impulsivity is linked to a more pronounced frontal dysfunction in adult ADHD subjects.

## Introduction

Attention-deficit hyperactivity disorder (ADHD) is a neurodevelopmental disorder, which is characterized by its core symptoms of developmentally inappropriate levels of inattention and/or hyperactivity and impulsivity (American Psychiatric Association 2013). The presence of ADHD symptomatology puts a high burden on social and academic functioning (Barkley 2002), and is associated with lower quality of life (Pulay et al. 2016). Meta-analyses and epidemiological studies reported a 2.5–4% prevalence of adult ADHD (Simon et al. 2009; Polanczyk et al. 2015; Fayyad et al. 2017), which underlines the importance of studies focusing on understanding adult ADHD.

Impulsivity, broadly defined as action without foresight, is an important component of ADHD at the behavioral level (Winstanley et al. 2006). However, the term ‘impulsivity’ covers a diversity of behavioral phenomena. To be able to study the behavioral problems observed in ADHD, the umbrella term needs to be further elucidated. Behavioral paradigms measuring impulsivity can be divided into two categories: those measuring impulsive choice/decision-making, and those measuring impulsive action/motoric impulsivity. The latter, impulsive action can be defined as the inability to refrain from making a response, and this impulse control is described as an active inhibitory mechanism (Winstanley et al. 2006).

The influential theoretical model of Barkley (Barkley 1997) suggests that inhibitory control is impaired in ADHD, and that this deficit is at the core of the disorder, as it underlies the diversity of executive difficulties present in ADHD (Grane et al. 2016). Based on this model, the identification of the factors behind the inhibitory control deficit in ADHD has received considerable attention in the literature.

To delineate inhibitory control in an experimental setting, Go/NoGo paradigms, which rely on the ability to suppress or withhold a prepared, but not yet initiated response, are commonly applied (Johnstone et al. 2013; Woltering et al. 2013). Based on these paradigms, a body of evidence has accumulated showing that inhibitory control deficits are present in children (Losier et al. 1996; Oosterlaan and Sergeant 1998; Schachar et al. 2000) and adolescents and adults with ADHD (for a review see Johnstone et al. 2013; Kaiser et al. 2020). However, while understanding the disorder on the behavioral level is important, the underlying neurobiological basis of inhibitory deficits needs to be further explored.

To do so, electrophysiological measures, such as high-density (64 channels and above) electroencephalography (EEG) and event-related potential (ERP) paradigms are increasingly applied. Through their high time resolution, they have the advantage of being able to capture the exact time course of distinct neurophysiological events during the applied paradigms within the millisecond range (Fallgatter and Strik 1999).

During the Go/NoGo paradigm, cancelation of the prepared behavioral response is necessary when an infrequent NoGo signal is presented. At the electrophysiological level, the NoGo signal elicits a pronounced ERP component, the P3. The NoGo P3 is thought to reflect the activity of multiple cortical locations, including the frontal cortex and the anterior cingulate and has been proposed to index the evaluation of the inhibitory process and its behavioral outcome (Fallgatter et al. 1997; Bruin et al. 2001; Huster et al. 2013). The P3 ERP component is a positive waveform typically measured between 300 and 600 ms post-stimulus with a fronto-central scalp distribution (e.g., Pfefferbaum et al. 1985; Johnstone et al. 2007).

Several studies have documented differences between individuals with and without ADHD for the NoGo P3 component and found significantly lower NoGo P3 amplitudes in children and adolescents with ADHD compared to controls over central scalp regions during auditory and visual response-control tasks (e.g., Fallgatter et al. 2004; Wiersema et al. 2006). A recent meta-analysis by Kaiser and colleagues (Kaiser et al. 2020) aimed to find the most robust neurophysiological deviations in terms of task-related ERPs in individuals with ADHD during the time course of cognitive processing. They reported that individuals with ADHD show smaller NoGo P3 amplitudes (and longer NoGo P3 latencies) compared to controls without ADHD and argue that their findings support the idea that the P3 component constitutes one of the most sensitive ADHD biomarkers.

While previous studies have primarily focused on the amplitude and latency of the P3 ERPs (Szuromi et al. 2011), an emerging body of literature indicates that in addition to these measures, the topography of this component contains critical information as well (Fallgatter et al. 2000). An important measure of scalp topography is the global field power (GFP), which provides a precise characterization of the spatiotemporal distribution of the brain’s electrical activity. In particular, it quantifies the amount of activity in terms of spatial standard deviation at each time point, considering all data from all recording electrodes, resulting in a reference-free, global descriptor of the potential field (Skrandies 1990).

Importantly, in addition to GFP, Fallgatter and colleagues (Fallgatter et al. 1997) have proposed the NoGo anteriorization index (NGA) as a standard neurophysiological measure of scalp topography for cognitive response control, indexing an overall shift of the brain’s electrical activity in anterior direction towards the prefrontal areas. The NGA is a parameter derived from mathematical data reduction relying on the entire information gained from the multichannel ERP registrations (Fallgatter and Strik 1999). Specifically, NGA is a comparison of EEG topographical maps between NoGo and Go ERPs; therefore, it reflects the brain electrical differences associated with the NoGo and the Go trials (Fallgatter and Strik 1999; Nash et al. 2013). Research consistently shows a more anterior located P3 topography in NoGo compared to Go trials (Fallgatter and Strik 1999; Fallgatter et al. 2005; Nash et al. 2013). This forward-shift or “anteriorization” of the NoGo P3 is believed to be based on a strong electrical activity of the anterior cingulate cortex (ACC) during the NoGo condition (Fallgatter et al. 2002). Higher NGA values are thought to reflect increased frontal activation recruited to control or inhibit the prepotent motor response.

While P3 NGA has been shown to be reduced in patients characterized by “disinhibition”, such as (childhood and adult) ADHD (and schizophrenia) (Fallgatter et al. 2004, 2005; Dresler et al. 2010), a limitation of the current literature is the paucity of data on clinically diagnosed adult ADHD patients, including detailed information on clinical characteristics such as pharmacological treatment and behavioral measures; and the lack of knowledge regarding the association of these measures with NGA. Additionally, previous studies are deficient in terms of EEG recordings obtained with high spatial resolution, which may be essential when we want to investigate brain activity with a high topographical accuracy (Gevins et al. 1994).

In the current study, we aimed to address these gaps of the current literature and to delineate the neurobiological basis of dysfunctional inhibitory processes by investigating the NoGo response in patients with adult ADHD and healthy controls. We also wanted to assess how neurobiological alterations are related to the clinical symptoms and behavioral measures of ADHD. Based on literature indicating that ADHD patients are characterized by a reduced P3 NGA, we hypothesized that the P3 topographical distribution would be altered, with less NoGo anteriorization in adult ADHD patients as compared to controls. We also expected higher impulsivity to be associated with reduced anteriorization of the P3 brain potential in the Go/NoGo task applied in our study.

## Methods

### Participants

A total of fifty-one subjects were included in the current study: 26 subjects with ADHD and 25 healthy controls. Healthy controls were matched to the patients by age (± 5 years), gender and level of education. Adult ADHD patients were recruited from the Adult ADHD Outpatient Clinic of the Department of Psychiatry and Psychotherapy, Semmelweis University, Budapest, Hungary. Controls were recruited from the local community, clinical staff, and their relatives.

Inclusion criterion for the patient group was a diagnosis of ADHD persisting into adulthood, established by an experienced psychiatrist. All diagnoses were made in adulthood. The psychiatric evaluation consisted of (1) a structured interview for evaluating current and retrospective childhood DSM-IV-TR ADHD symptoms; (2) semi-structured and open interviews assessing background information, developmental data, functional impairment, psychiatric comorbidity; (3) medical history data obtained from medical documentation and close family members, and (4) self-rated questionnaires.

Exclusion criteria for all participants included a history of severe neurological or somatic disorder or severe head trauma. Controls were also excluded in case of positive neurological or psychiatric history. No exclusions were made based on these criteria. The 90-item Symptom Checklist (SCL-90R) was used to select controls with no current psychiatric comorbidity (Derogatis and Cleary 1977). No control subjects were excluded based on SCL-90R scores.

All participants gave written informed consent for the study. The study received approval by the Institutional Research Ethics Committee of Semmelweis University and was conducted according to the Declaration of Helsinki.

### Stimuli and procedure

Participants performed the task in a dimly lit, sound-attenuated room between 10 a.m. and 2 p.m. They were asked not to take their medication (if any) the morning before the investigation. The computer screen for stimuli was placed at a viewing distance of approximately 50 cm. The applied Go/NoGo paradigm was previously used and described in detail (Durston et al. 2002; Papp et al. 2020). Similar to the prior study developed for children with ADHD by Durston and colleagues, (Durston et al. 2002), characters from the Pokémon cartoon series were used as visual stimuli. Participants were instructed to respond with pressing a button when a Go picture appeared on the screen and to withhold responding in case of rare NoGo trials. Furthermore, participants were instructed to respond as quickly and accurately as possible. The task consisted of 5 runs, 57 pictures in each run. All pictures were presented for 1 s and were followed by an interstimulus interval of 3 s. Each run consisted of 75% Go trials, and 25% NoGo trials. During the task, different types of NoGo trials were presented in a pseudorandom order; NoGos were preceded either by 1, 3, or 5 Go trials. Similarly to the original study (Durston et al. 2002) the task difficulty was manipulated by parametrically varying the number of Go trials preceding a NoGo trial.

### Clinical measures

To assess ADHD symptom severity the Conners’ Adult ADHD Rating Scale—Self-Report: Long Version (CAARS) was applied (Conners et al. 2011). To describe symptom severity on ADHD symptom domains, total scores of all CAARS subscales (Inattention, Hyperactivity, Impulsivity and Problems with Self-concept) were calculated in both study groups.

### EEG recording and preprocessing

EEGs were recorded by the 128-channel Biosemi Active Two system (Biosemi Inc., Amsterdam, Netherlands) at a sampling rate of 1024 Hz. A band-pass filter of 0.5–70 Hz was applied. Electro-oculogram (EOG) was recorded for monitoring eye movements for artifact identification and rejection. Besides eye movements, epochs with a voltage exceeding ± 90 μV on any EEG or EOG channel were excluded applying automatic artifact rejection criteria. Data were stored and analyzed off-line using the Electromagnetic Source Signal Imaging (EMSE) Suite and the Statistical Analysis System (SAS 9.4) software. The stimulus-locked data were segmented into epochs of 700 ms, including 200 ms before stimulus and 500 ms after stimulus. The threshold cut-off was 50 for the required minimum number of usable segments for the ERP analyses. Only correct trials were included in the current analyses. The stimulus-locked segments were baseline-corrected using a 200-ms pre-response window and averaged to obtain the ERP waveforms for each subject and each condition (Go/NoGo).

### EEG data processing

Our EEG data analyses followed a 2-step procedure, including (1) the determination of global field power as a function of time in relation to the stimulus onset to identify a data-driven time window of interest; (2) computation of the amplitude centroid to measure the NoGo anteriorization of the ERP NoGo responses relative to the Go responses in the specified time window of interest. Below, a detailed description of the two steps is provided.

**Fig. 1:**
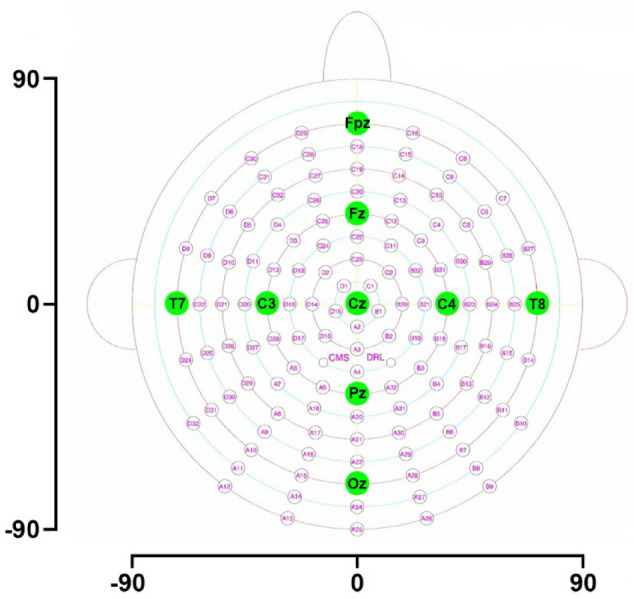
Planar projection of the Biosemi 128-electrode array on a head shape. View from above: upper is anterior, left is left side of the head. Locations of the centroids were computed from average reference maps and quantified by a coordinate system resulting from the planar projection of the BioSemi electrode array onto a circular angular grid, extending from 90 to – 90 degrees, both anterior–posterior and central to lateral directions. The axes with the coordinate values are displayed next to the head shape

Step 1. Determination of global field power. Following the procedures of Fallgatter and colleagues (Fallgatter et al. 1997), in the initial step of our analyses we determined the GFP of the difference between the NoGo and the Go ERPs at each time point for the 500-ms period following stimulus onset. GFP has been shown to represent a robust measure of the spatiotemporal characteristics of brain activity, corresponding to the spatial standard deviation of the electrical potentials recorded at each time point across all electrodes (Lehmann 1980). As pointed out by Fallgatter et al. (Fallgatter et al. 1997), the GFP curve of the difference map takes into account both differences in field strength and topography of the ERP map series. Similar to a landmark study (Lehmann 1980), the GFP curve was used for the component segmentation of the ERPs. In particular, based on data from all participating subjects in the study, we identified the GFP maximum in the P3 time window of interest identified by Fallgatter and colleagues (Fallgatter et al. 1997) for the Go/NoGo difference maps. The time points of the minima preceding and following the maximum were selected as the borders of the respective P3 segment (see also Fig. 1, in the Results).

Step 2. Computation of the amplitude centroid to measure the NoGo anteriorization (NGA). Within the time window identified in Step 1, we computed the centroids based on the amplitude and topographical configuration of the respective map (Lehmann 1980, 1987; Lehmann and Skrandies 1984). The centroids represent the amplitude-weighted locations of the positive and the negative part of the topographical distribution of the brain’s electrical activity (i.e., the “center of gravity” of the brain activity) (Lehmann and Skrandies 1984; Lehmann 1987). Locations of the centroids were computed from average reference maps and were quantified by a coordinate system resulting from the planar projection of the BioSemi electrode array onto a circular angular grid, extending from 90 to -90 degrees, both anterior–posterior and central to lateral directions (Fig. 1). Higher positive value in anterior–posterior direction means more pronounced anteriorization of the scalp topographical distribution, i.e., a greater NGA.

To examine the robustness of the findings, we conducted analyses by computing the NGA (centroid) measure for the full time window (i.e., 280 to 380 ms post-stimulus, see later); and for the peak GFP (in a 20-ms time interval around the GFP maximum of interest), since the latter (peak GFP) may delineate changes that are associated more specifically with the event of interest (i.e., at the most pronounced manifestation of P3 in time). Furthermore, we also determined the NGA (centroid) measure separately, based, respectively, on the full set of 128 electrodes; and on the anterior midline electrodes, since these sensors are considered to represent the best established region of interest for the NoGo P3 ERP component. The effect size for the group difference in NGA was characterized in terms of the Cohen D measure (Cohen 1988).

The statistical analyses were based on random-regression hierarchical linear modeling (HLM) (Gibbons et al. 1988; Bryk and Raudenbush 1992). In separate analyses, repeated measurements of the GFP amplitude (in microvolt-squares) in the P3 ERP time window of interest served as dependent variable in the HLM. Study group (between-subjects factor) was the principal independent variable of interest. Time (sampling point in the component window, relative to stimulus onset) was included in the analysis as a within-subject factor. We also included gender and age as independent variables in the analyses to control for their confounding effects. A first-order autoregressive moving average correlation matrix among the sampling points was specified in the HLM model. In subsidiary analyses, we examined the effect of several clinically important variables, including medication status, use of psychostimulants, measures of psychopathology as indexed by the subscales of the CAARS, and behavioral indices such as the reaction time. In separate analyses, we introduced these variables as additional covariates in the HLM, thereby incorporating a regression estimation into the General Linear Mixed Model. This analysis allowed us to estimate the NGA values for specific values of the covariates. To illustrate the sign and strength of the regression relationship within each group, for each covariate of interest (e.g., a subscale score on the CAARS) a low and high value (representing, respectively, the lower and upper quartile of the distribution) was selected to estimate the NGA. The Hochberg procedure was used for adjustment for multiple testing.

## Results

### Demographics and basic descriptive characteristics

The summary of basic demographic, behavioral and symptom severity characteristics of the study population is provided in Table 1. Age, gender and years of education were not significantly different between the ADHD and the control groups.

**Table 1:** Basic demographic and clinical characteristics of the study sample

Characteristics	ADHD (*N* = 26)	Control (*N* = 25)	*F*/Chi^2^	*p*
Male, *N* (%)	18 (69.23)	19 (76.00)	0.29^a^	0.5881
Affective comorbidity, *N* (%)	11 (42.31)	0 (0)	0	n.a.^c^
Stimulant medication, *N* (%)	9 (34.62)	0 (0)	0	n.a.^c^
Other medication, *N* (%)	3 (11.54)	0 (0)	0	n.a.^c^
Age, years (mean, SD)	28.92 (8.38)	27.28 (5.03)	0.71^b^	0.4022
Years of education (Mean, SD)	14.04 (2.54)	16.28 (1.59)	13.94^b^	0.0005
Conners’ Adult ADHD Rating Scale				
Total core symptom domain score (mean, SD)^h,i^	89.14 (22.14)	44.14 (26.20)	*F*_grp_ = 27.87, *p* < 0.0001 *F*_gender_ = 4.37, *p* = 0.0429	
Male	84.89 (20.48)	39.69 (24.26)		
Female	98.85 (24.31)	59.28 (29.65)		
Hyperactivity/Restlessness (Mean, SD)^h,i^	20.09 (4.76)	10.10 (6.22)	*F*_grp_ = 26.92, *p* < 0.0001 *F*_gender_ = 2.09, *p* = 0.1555	
Male^d,f^	19.28 (4.14)	9.47 (6.16)		
Female^e,g^	21.94 (5.87)	12.24 (6.65)		
Inattention/Memory problems (Mean, SD)^h,i^	23.74 (7.03)	11.12 (7.90)	*F*_grp_ = 22.96, *p* < 0.0001 *F*_gender_ = 1.44, *p* = 0.2371	
Male^d,f^	22.88 (6.84)	10.38 (7.77)		
Female^e,g^	25.71 (7.58)	13.68 (8.72)		
Impulsivity/Emotional problems (Mean, SD)^h,i^	17.59 (7.36)	9.07 (6.45)	*F*_grp_ = 11.41, *p* < 0.0016 *F*_gender_ = 1.79, *p* = 0.1881	
Male^d,f^	16.81 (6.81)	8.22 (6.55)		
Female^e,g^	19.38 (8.80)	11.98 (5.76)		
Problems with Self-concept (Mean, SD)^h,i^	10.13 (5.52)	4.77 (4.38)	*F*_grp_ = 7.65, *p* = 0.0085 *F*_gender_ = 8.64, *p* = 0.0054	
Male^d,f^	9.13 (4.98)	3.41 (2.94)		
Female^e,g^	12.43 (6.40)	9.40 (5.64)		
Reaction time, msec (mean, SD)^h,i^	505.94 (70.93)	505.58 (71.51)	*F*_grp_ = 0.14 *p* = 0.7150 *F*_gender_ = 1.47 *p* = 0.2314	
Male^d,f^	509.70 (54.86)	516.45 (72.93)		
Female^e,g^	496.80 (105.46)	472.98 (61.15)		
Correct answers, % (mean, SD)^h,i^	91.25 (11.49)	96.96 (4.03)	*F*_grp_ = 7.31, *p* = 0.0097 *F*_gender_ = 0.87, *p* = 0.3565	
Male^d,f^	0.93 (0.06)	0.97 (72.93)		
Female	0.87 (0.19)	0.98 (0.03)		

^a^Chi-square test, Chi-square

^b^ANOVA, *F*

^c^Comorbidity/medication was not present at the control group

^d^*N* = 18 for males in the ADHD group

^e^*N* = 8 for females in the ADHD group

^f^*N* = 19 for males in the control group

^g^*N* = 6 for females in the control group

^h^ANOVA with group effect (*F*_grp_), gender effect (*F*_gender_) and interaction effect between group and gender as independent variables, ^i^no significant interaction between group (grp) and gender (*p* > 0.1)

ADHD patients were characterized by significantly higher overall symptom severity as measured by the CAARS Total core symptom score compared to controls (*F* = 38.84, *p* < 0.0001). As expected, all four CAARS symptom factors were significantly higher in the ADHD group compared to healthy controls: Hyperactivity (*F* = 36.74, *p* < 0.0001), Inattention (*F* = 32.09, *p* =  < 0.0001), Impulsivity (*F* = 16.98, *p* = 0.0002) and Problems with self-concept subscales (*F* = 12.92, *p* = 0.0008). All of the adult ADHD patients belonged to the combined subtype of the disorder.

As for comorbidity, a total of 11 (42.3%) patients had another DSM-IV-TR psychiatric diagnosis, all of which were affective disorders (unipolar depression and anxiety). Personality disorders were not assessed in the current study. Psychopharmacological treatment was received in 46.2% of cases (*n* = 12). Nine patients received methylphenidate (34.6%); and 3 patients (11.5%) received antidepressants, with 1 of them receiving anxiolytics (for adjustment disorder). Regarding non-stimulant medication, bupropion was administered to 3 patients (combined with paroxetine in 1 patient and with clonazepam in another one).

Task performance speed was comparable in the two study groups (505.9 ms vs. 505.6 ms, *p* = 0.9862 in the ADHD and control group, respectively). ADHD patients performed significantly worse compared to control subjects (commission error rates were 8.8% in the ADHD and 3.0% in the control group, respectively).

### Electrophysiological results

**Fig. 2:**
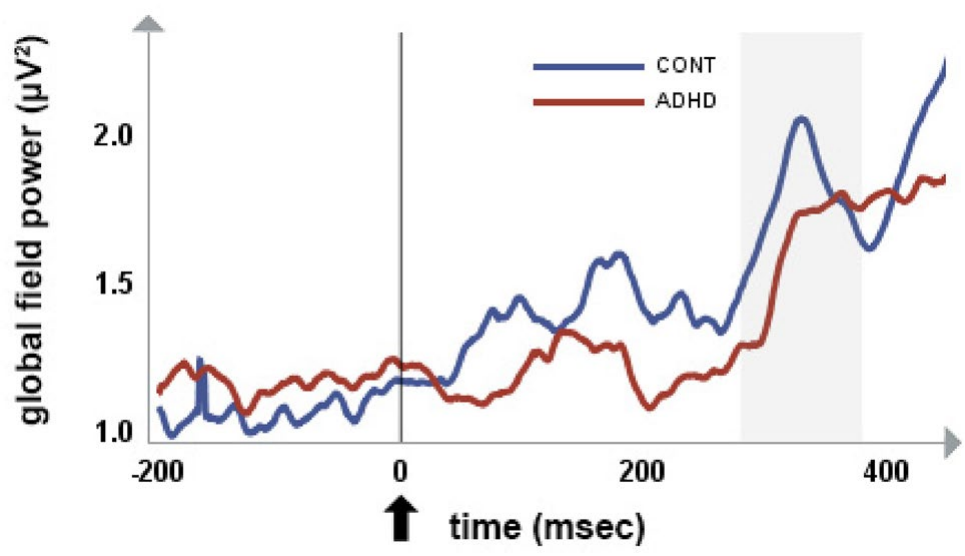
GFP difference waves in the ADHD and control groups, derived by subtracting the GFP for NoGo trials from the GFP for Go trials in each group. Shaded bands represent the analyzed 280–380-ms time window, in which the difference was significant (*F* = 66.62, *p* < .0001) between the ADHD and control groups. Stimulus onset was at 0 ms, indicated by the arrow

As described in the Methods, in the initial step of our analyses, the global field power of the difference between the Go and NoGo ERPs was calculated. As shown by Fig. 2, the GFP curve of the difference map showed a clear maximum at approximately 330 ms post-stimulus in the healthy control group, which we considered as our basic benchmark based on the empirical data. Since the respective data-driven segment surrounding the GFP peak enclosed the post-stimulus time window between 280 and 380 ms, this time frame (i.e., 280–380 ms) was chosen as the P3 window of interest in our study and was focused on in further analyses. We note that our results are rather similar to the GFP curve of the difference maps of Fallgatter and colleagues (Fallgatter et al. 1997), whose empirically determined time window in that study was between 277 and 434 ms.

Regarding the grand average of Go–NoGo GFP amplitudes in the two groups (Fig. 2), ADHD patients had significantly lower amplitudes compared to controls in the analyzed 280–380-ms time frame (*F* = 66.62, *p* < 0.0001). This result was independent of age and gender.

**Fig. 3:**
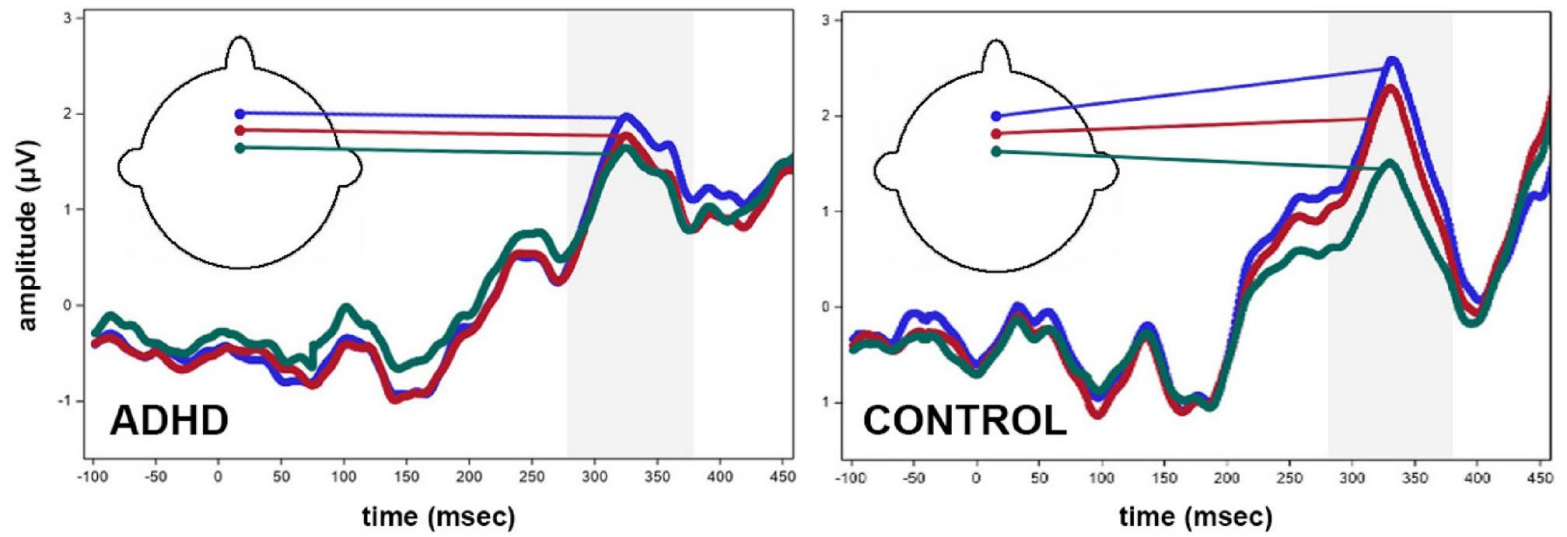
Waveforms for raw amplitude (μV) values for stimulus-locked ERPs on mid-anterior electrode sites (Fz, FCz in the International 10–20 System, and the midline electrode between them, the latter, labeled as C22 in BioSemi electrode layout) for the NoGo condition in ADHD patients and control subjects. Shaded bands represent the analyzed 280–380-ms time window, in which the difference was significant (*F* = 66.62, *p* < .0001) between the ADHD and control groups

In order to visualize the NoGo anteriorization in terms of the original ERP curves, we depicted the grand average ERPs for the ADHD and the control groups from three mid-anterior electrodes (Fz, FCz in the International 10–20 System, and the midline electrode between them, the latter, labeled as C22 in BioSemi layout) that are commonly focused on for the investigation of the NoGo P3 (Fig. 3). As shown by Fig. 3, an anteriorization effect was observable in both groups in the 280–380-ms time frame (i.e., larger P3 amplitudes were observable at more anterior electrodes in both groups). However, the figure also indicates that the anteriorization of the NoGo P3 component was considerably more prominent in the control as compared to the ADHD group.

Using HLM analyses, we examined whether the statistical measure of the NGA, as described by the topographical centroid value, differed between the ADHD and control groups within the whole identified P3 time frame (full 280–380-ms window). Our results indicate that the NGA was significantly less pronounced in ADHD patients than in healthy controls (32.68 vs. 40.18, *F* = 60.76, *p* < 0.0001), with a Cohen D effect size of 1.56 for the group difference. We also investigated whether the difference in NGA between patient and control groups is also observable at the peak GFP, defined as a P3 time window of 20 ms around the GFP maximum. Our analyses for the GFP peak yielded similar findings to those observed for the full P3 time window; ADHD patients had a significantly lower NGA than controls: 5.77 vs. 13.40, *F* = 11.14, *p* = 0.0016), with a Cohen D effect size of 0.68 for the group difference. Detailed results are shown in Table 2*.*

**Table 2:** ADHD vs. control: Group differences in anteriorization

NoGo anteriorization (NGA) in the P3 time window	Group (estimated mean, SE)		*F*	*p*
	ADHD	Control		
NGA at GFP peak (defined as 20 ms around GFP maximum)	5.77 (1.60)	13.40 (1.63)	11.14	0.0016
NGA in full P3 window (280–380 ms), enclosing the period between GFP maximum and neighboring minima	32.68 (0.74)	40.18 (0.62)	60.76	< .0001

In our further analyses, we focused on the GFP peak within the P3 time frame, since, as described in the Methods, this may delineate changes at the most pronounced manifestation of P3 in time and report our results accordingly.

The difference in NGA between the ADHD and control group remained significant after correcting for medication status, even though medication use per se was associated with increased NGA (2.50 of unmedicated vs. 9.57 of medicated patients *F* = 4.52, *p* = 0.0441). In addition to using medication status as covariate in the analyses, we performed a sensitivity analysis by comparing the NGA results between ADHD unmedicated and ADHD medicated groups against control subjects. We had the following findings: NGA at GFP peak of the ADHD unmedicated group (*n* = 14) was 2.50 (SE 2.18), while NGA of the ADHD medicated group (*n* = 12) was 9.57 (SE 2.36). Hence, the direct comparison between the two ADHD groups and the control group revealed consistent results with the approach we used previously with medication as a covariate: ADHD patients had lower NGA than controls. The difference between the ADHD unmedicated and control groups was significant (*p* = 0.0002), similar to the difference between the two ADHD groups (*p* = 0.0325), while the difference between the ADHD medicated and control groups did not reach significance (*p* = 0.1877).

The use of stimulant medication in itself was also associated with a numerically more pronounced NGA, with the difference between NGA of patients receiving stimulants compared to those not taking stimulant medication approaching marginal significance (*F* = 2.55, *p* = 0.1171). When we excluded ADHD patients who were taking stimulant medication (*n* = 9), the NGA was still significantly less pronounced in ADHD patients comp**a**red with healthy controls (*p* < 0.0001). The presence of comorbidity did not have a significant effect on our results.

Associations between NGA and ADHD symptom severity, as measured by the CAARS Hyperactivity, Impulsivity, Inattention and Problems with Self-Concept subscales were examined using the data of the ADHD group. The analysis was conducted for the mid-anterior electrodes (Fz, FCz and the electrode between them) to capture the changes at the most specific scalp region within the P3 time window (280–380 ms).

After corrections for multiple comparisons, we found a significant inverse relationship between NGA values and CAARS Impulsivity scores (*F* = 9.39, *p* = 0.0059): higher Impulsivity scores were associated with lower NGA values. The relationship between NGA anteriorization and CAARS symptom dimensions is shown in Table 3. In our subsidiary analyses we examined the relationship between symptom severity and NGA based on all 128 electrodes in the selected P3 time frame. The results were similar to those we found for the mid-anterior electrodes (i.e., a significant inverse relationship between NGA and CAARS Impulsivity score). We note, however, that when patients with stimulant medication were excluded from the analysis (*n* = 9) the results did not reach significance in the limited sample (*F* = 2.19, *p* = 0.1623), even though the direction of the relationship between NGA values and CAARS Impulsivity scores remained the same.

**Table 3:** Relationship between symptom severity (as measured by CAARS subscales) and NoGo anteriorization amplitudes on mid-anterior electrode sites between 280 and 380 ms

CAARS domain	NoGo anteriorization (estimated mean, SE) at low and high symptom severity on the four CAARS subscales		Difference *F* (*P*)^a^
	Low	High	
Hyperactivity	1.40 (2.44)	6.64 (7.38)	0.94 (0.3437)
Impulsivity	– 5.57 (2.39)	– 16.21 (5.45)	9.39 (0.0059)
Inattention	– 0.09 (1.71)	2.62 (4.32)	0.55 (0.4683)
Self-concept	2.95 (4.93)	6.25 (9.45)	0.50 (0.4879)

Notes: Low and high values of the CAARS subscales were defined as a value representing the lower and upper quartile, respectively, of the empirical distribution of the subscale scores

^a^Corrections for multiple comparisons were applied

Besides the association between NGA and symptom severity, we also examined the relationship between reaction time and NGA in connection with impulsivity (as the latter symptom’s dimension reached significance in the analyses). The latter analyses were conducted using the data from patients with ADHD. Analysis of covariance was performed in the ADHD group to investigate the joint impact of behavioral variables and impulsivity, as measured by the CAARS Impulsivity subscale, on the extent of NoGo anteriorization. A separate analysis was conducted for each of the two behavioral measures (reaction time and number of correct responses). The results indicated a statistically significant interaction between the behavioral measures and impulsivity.

After correction for multiple comparisons, we found that the interaction between reaction time and impulsivity was significant on NGA (*F* = 22.78, *p* = 0.0002). Significant changes occurred in association with high impulsivity scores. Specifically, as shown by the upper part of Table 4, lower reaction time (fast response) with high impulsivity (high score on the CAARS Impulsivity domain) was associated with the lowest NGA*.* The interaction between the rate of correct responses and impulsivity was also significant on NGA (*F* = 65.3, *p* < 0.0001). Post hoc analyses indicated that low rate of correct responses (more commission errors) with high impulsivity (high score on the CAARS Impulsivity domain) was associated with the most pronounced decrease of NGA in the ADHD group *(lower part of *Table 4*).*

**Table 4:** Relationships between reaction time, number of correct responses, impulsivity and NGA

	Impulsivity	
	Low	High
Reaction time		
Fast^a^	– 16.36 (3.43)	– 65.74 (8.41)
Slow^b^	– 3.61 (4.93)	– 5.16 (10.70)
Number of correct responses		
Low^c^	– 5.65 (2.41)	– 42.74 (5.28)
High^d^	– 12.02 (2.63)	– 27.53 (6.12)

Notes: The values in the table represent NGA estimates and standard errors (SE) at low and high values of the two covariates in the analyses (i.e., behavioral measure, impulsivity). Low and high values of the covariates were defined as a value representing the lower and upper quartile, respectively, of the empirical distribution of the covariates

^a^NGA estimate (SE) for reaction time at the upper quartile (i.e., 75%) of the reaction time distribution

^b^NGA estimate (SE) for reaction time at the lower quartile (i.e., 25%) of the reaction time distribution

^c^NGA estimate (SE) for number of correct responses at the upper quartile (i.e.,75%) of response accuracy distribution

^d^NGA estimate (SE) for number of correct responses at the lower quartile (i.e.,25%) of response accuracy distribution

## Discussion

We investigated the behavioral and electrophysiological correlates of inhibitory processing during a visual Go/NoGo study in subjects with adult ADHD and healthy controls.

We found that adult ADHD patients had a significant reduction in the Go–NoGo GFP in the P3 latency range compared to control subjects. Altered topographical distribution and less spatial variation of ADHD subjects suggest a complex neurophysiological dysfunction present in the disorder.

Specifically, our main finding that adult ADHD patients are characterized by a reduced P3 NGA is consistent with a prior study, which reported lower NGA in children with ADHD (Fallgatter et al. 2004). Similarly, the finding is congruent with previous research, which reported that reduced NGA values were present in adult patients with ADHD-related psychopathology during childhood, i.e., persisting ADHD (Fallgatter et al. 2005). Furthermore, past research including a large number of adult ADHD patients found a tendency of lower NGA values in patients as compared to controls (Dresler et al. 2010). In contrast to previous studies, however, we included adult ADHD subjects with an established ADHD diagnosis and had an EEG spatial resolution superior to preceding publications that used low sensor density / sparse spatial sampling (Fallgatter et al. 2004, 2005; Dresler et al. 2010). Therefore, our results of a reduced P3 NGA are in line with and further extend previous findings, in terms of the enhanced spatial resolution and a thorough clinical diagnosis of adult ADHD.

While NGA is thought to reflect the mechanisms of prefrontal response control (Fallgatter and Strik 1999; Dresler et al. 2010), some might argue that reduced NoGo anteriorization as compared to healthy controls is instead a neural marker of a general executive dysfunction. Since we found that NGA was strongly associated with impulsivity, our findings suggest that NGA is more likely to reflect a more specific impairment of the inhibitory control subdomain rather than that of general executive functions. Nash and colleagues (Nash et al. 2013) demonstrated NGA to be a predictor of self-control in a social exchange game, i.e., greater NoGo anteriorization was associated with better self-control. This finding is also consistent with the notion that NGA is a reflection of inhibitory control.

In our sample, the behavioral performance as measured by commission errors was significantly worse in ADHD patients as compared to controls, while task performance speed was similar in the two study groups. Both findings are in line with prior literature reporting adult ADHD patients making significantly more commission errors than controls (e.g., Hervey et al. 2004; Dresler et al. 2010; Woltering et al. 2013), while not being significantly different from healthy controls in reaction time (e.g., Wiersema et al. 2006; Prox et al. 2007; Woltering et al. 2013; Grane et al. 2016). However, it is important to note that prior results are inconsistent both in terms of task performance and reaction time: some studies report similar (not significantly different) task performance of adult ADHD and control subjects in visual Go/NoGo studies (e.g., Wiersema et al. 2006; Prox et al. 2007; Helenius et al. 2011). As for reaction time, in some studies, adult ADHD patients were found to be significantly slower compared to controls (e.g., Valko et al. 2009; McLoughlin et al. 2010). The currently available clinical findings, therefore, remain equivocal. This might arise from the fact that there are important differences among the Go/NoGo studies regarding study size, applied tasks, task instructions and the clinical characteristics of study participants. For example, a former meta-analysis (Bálint et al. 2009) concluded that adult ADHD subjects exhibit significantly poorer functioning than healthy controls on complex tasks of attention (such as the NoGo task in the current study), and the degree of impairment is greater in males. This conclusion is consistent with the results of the current study, which are based on predominantly male population with adult ADHD using a Go/NoGo paradigm.

Our finding of a lower NGA is in agreement with the notion of inhibitory control problems and frontal lobe dysfunctions in ADHD. Research on neuropsychological tests provide evidence of executive dysfunctions related to the prefrontal cortex present in ADHD (Pievsky and McGrath 2017), findings which are supported by neuroimaging studies that described lower brain activation in frontal regions of adult ADHD patients using various task paradigms focusing on inhibition and inattention in the disorder (Hart et al. 2013; Lukito et al. 2020). In order to achieve a more thorough understanding of NGA alteration in ADHD, we applied various analytical approaches to investigate the anteriorization. We found that our results of a lower NGA in adult ADHD patients were observable regardless of the applied analysis. These data, taken together, provide convergent evidence for the alteration of NGA and strengthen the validity of the findings.

The possibility that lower NoGo anteriorization is a result of altered neurodevelopment in ADHD requires consideration. Since an emerging body of neuroimaging studies shows that the human brain has a high plasticity (Jäncke 2009) throughout the lifespan (Draganski et al. 2004, 2006; Draganski and May 2008), the lack of (or lessening of) or impairment of anteriorization could be the result of an altered neurodevelopmental pathway of prefrontal cortex maturation in ADHD (Shaw et al. 2007).

We investigated the role of medication on NGA and found that the use of stimulant medication was associated with a more pronounced NGA, i.e., the NGA values of patients taking methylphenidate were closer to the values of controls. This electrophysiological finding is in line with the observed behavioral effect of stimulants on impulsivity (e.g., Jensen et al. 2001). While EEG correlates of methylphenidate administration were reported earlier (e.g., Loo et al. 2004; Skirrow et al. 2015; Rubinson et al. 2019), the NGA was not in the focus in those studies. Therefore, it is important to note that while our primary goal was to investigate anteriorization deficits in our group of adult ADHD patients in a specific task condition, the finding of the “normalization” effect of methylphenidate on NGA highlights the value of this parameter. With respect to potential medication effects on NGA, it is of note that atypical antipsychotics were reported to “stabilize” or even increase NoGo anteriorization in patients with schizophrenia spectrum disorders (Ehlis et al. 2007), while also having a more favorable impact on cognitive functioning in schizophrenia patients than typical antipsychotics (e.g., Guilera et al. 2009). Since NGA is considered to be a neurophysiological correlate of response control (Fallgatter et al. 2002), our findings that clinically more severe impulsivity is linked with a lower NGA outline a connection between clinical characteristics, executive functions, and electrophysiological measures.

To have a deeper understanding of the relations between NGA, clinical characteristics, behavioral measures, and a combination of these factors, we examined the relationship between a) reaction time, impulsivity and NGA and b) number of correct responses, severity on the CAARS Impulsivity subscale and NoGo anteriorization. Our results highlight that the most the prominent alterations in NGA are linked to a covariation of certain behavioral and clinical measures, such as fast reaction times and high error rates combined with high impulsivity in ADHD subjects. To our knowledge, the current study is the first to describe the relationship between behavioral and clinical variables including ADHD severity and NoGo anteriorization, making it possible to connect specific ADHD symptoms and their severity to NGA, a highly reliable electrophysiological correlate of prefrontal/cognitive response control (Fallgatter et al. 2004). As it has been suggested, a core deficit in inhibition control might account for executive dysfunction in ADHD, which underlies most of the dysfunctional behaviors associated with this disorder (Fallgatter et al. 2004). The use of high-density EEG to study the NGA may provide a better understanding of inhibitory dysfunction in ADHD.

Additionally, it is important to note that the large effect size that we found for NGA makes it a good candidate for a potential biomarker for ADHD. Furthermore, the fact that the P3 NoGo anteriorization is a reliable and simple-to-use measure may pave the way for a clinical application of novel neuromodulation treatments based on NGA, in order to modify altered neural activity in ADHD. These treatments, including neurofeedback or non-invasive brain stimulation are increasingly being viewed as promising in targeting the key neurobiological abnormalities associated with ADHD (Rubia 2022).

Limitations of our study include the relatively small sample size, which—while similar to the sample sizes used in other ERP studies (Fallgatter et al. 2005; McLoughlin et al. 2010; Grane et al. 2016)—did not allow for detailed analyses regarding ADHD subtypes. Also, while the ADHD and control groups significantly differed regarding task performance, the overall task performance was good. Therefore, future studies should apply tasks with a greater level of difficulty in order to achieve better group separation. Additionally, the task instruction we used (i.e., the prompt to respond as quickly and accurately as possible), might have resulted in some participants focusing on speed and some on accuracy, leading to different behavioral outcomes. A further study limitation is that approximately half of the patients received medication, and about one-third had comorbidities. However, the inclusion of medication and comorbidity status in the analyses did not influence the main results. Finally, while common in the literature, recruitment of controls from clinical staff and their relatives can also be considered as a limitation in terms of generalizability. Overall, despite the limitations, the decreased NGA observed in our study underlines the importance of inhibitory control dysfunction in adult ADHD at the neurophysiological level and requires further exploration.

## Data Availability

The datasets generated during and/or analyzed during the current study are available from the corresponding author on reasonable request.
